# Tissue Transglutaminase Promotes Early Differentiation of Oligodendrocyte Progenitor Cells

**DOI:** 10.3389/fncel.2019.00281

**Published:** 2019-07-02

**Authors:** Nathaly Espitia Pinzon, Hanneke van Mierlo, Jenny C. de Jonge, John J. P. Brevé, John G. J. M. Bol, Benjamin Drukarch, Anne-Marie van Dam, Wia Baron

**Affiliations:** ^1^Department of Anatomy and Neurosciences, Amsterdam UMC, Vrije Universiteit Amsterdam, Amsterdam Neuroscience, Amsterdam, Netherlands; ^2^Department of Biomedical Sciences of Cells & Systems, Section Molecular Neurobiology, University of Groningen, University Medical Center Groningen, Groningen, Netherlands

**Keywords:** tissue transglutaminase, oligodendrocyte progenitor cells, multiple sclerosis, remyelination, astrocytes, differentiation

## Abstract

Demyelinated lesions of the central nervous system are characteristic for multiple sclerosis (MS). Remyelination is not very effective, particular at later stages of the disease, which results in a chronic neurodegenerative character with worsening of symptoms. Previously, we have shown that the enzyme Tissue Transglutaminase (TG2) is downregulated upon differentiation of oligodendrocyte progenitor cells (OPCs) into myelin-forming oligodendrocytes and that TG2 knock-out mice lag behind in remyelination after cuprizone-induced demyelination. Here, we examined whether astrocytic or oligodendroglial TG2 affects OPCs in a cell-specific manner to modulate their differentiation, and therefore myelination. Our findings indicate that human TG2-expressing astrocytes did not modulate OPC differentiation and myelination. In contrast, persistent TG2 expression upon OPC maturation or exogenously added recombinant TG2 accelerated OPC differentiation and myelin membrane formation. Continuous exposure of recombinant TG2 to OPCs at different consecutive developmental stages, however, decreased OPC differentiation and myelin membrane formation, while it enhanced myelination in dorsal root ganglion neuron-OPC co-cultures. In MS lesions, TG2 is absent in OPCs, while human OPCs show TG2 immunoreactivity during brain development. Exposure to the MS-relevant pro-inflammatory cytokine IFN-γ increased TG2 expression in OPCs and prolonged expression of endogenous TG2 upon differentiation. However, despite the increased TG2 levels, OPC maturation was not accelerated, indicating that TG2-mediated OPC differentiation may be counteracted by other pathways. Together, our data show that TG2, either endogenously expressed, or exogenously supplied to OPCs, accelerates early OPC differentiation. A better understanding of the role of TG2 in the OPC differentiation process during MS is of therapeutic interest to overcome remyelination failure.

## Introduction

Multiple sclerosis (MS) is a chronic inflammatory, demyelinating disease of the central nervous system (CNS) most often starting in young adults (Hafler, 2004; Compston and Coles, 2008). Clinical symptoms include impairment of sensory, cognitive and motor functions (Noseworthy et al., 2000). The pathology of MS is characterized by focal lesions of demyelination and infiltration of leukocytes into the CNS due to a functionally impaired blood-brain-barrier (BBB) (Ortiz et al., 2014). Resident immune cells in the CNS, i.e., microglia, become activated and secrete cytokines and chemokines that recruit more immune cells into the CNS (Huang et al., 2000; Sellebjerg and Sørensen, 2003). Consequently, axons are demyelinated and oligodendrocytes (OLGs) are eliminated from MS lesions (Reynolds et al., 2011). To overcome the damage in the CNS, a regenerative response is initiated that involves recruitment of oligodendrocyte progenitor cells (OPCs) toward the demyelinated areas by factors, primarily produced by lesional microglia and astrocytes (Carroll and Jennings, 1994; Glezer et al., 2006). OPCs subsequently interact with demyelinated axons and differentiate into mature myelinating OLGs (Franklin et al., 1997; Sim et al., 2002; Rhodes et al., 2006). The remyelination process in MS lesions is, however, not very effective, in particular at later stages of the disease (Compston and Coles, 2008; Franklin and ffrench-Constant, 2008; Kuhlmann et al., 2008; Goldschmidt et al., 2009; Kotter et al., 2011), which results in the chronic neurodegenerative character of this disease with worsening of symptoms (Lublin et al., 2014). Even though recently it has been shown that mature OLGs can contribute to remyelination (Jäkel et al., 2019; Yeung et al., 2019), there is a clear role of OPCs to differentiate into myelin producing OLGs to overcome demyelination. However, in the progressive stage of the disease, approximately 70% of MS lesions that remain demyelinated contain OPCs, suggesting a failure of OPC maturation in the majority of MS lesions. The other 30% of the demyelinated lesions contain few or no OPCs, indicating failure of proliferation and/or migration (Lucchinetti et al., 1999; Kuhlmann et al., 2008; Fancy et al., 2010; Maki et al., 2013).

Tissue Transglutaminase (TG2) is a Ca^2+^-dependent 78 kD multifunctional enzyme expressed in the CNS, including neurons, astrocytes, microglia and OPCs (Fesus and Piacentini, 2002; van Strien et al., 2011c; Numinskaya and Belkin, 2012; Espitia Pinzón et al., 2014). Its functions include the cross-linking of proteins (Aeschlimann and Thomazy, 2000), the binding to fibronectin, thereby promoting cell-matrix interactions (Akimov and Belkin, 2001) and the binding and hydrolysis of GTP enabling transmembrane signaling in cells (Nakaoka et al., 1994). These functions are encoded for by different structural molecular domains of the enzyme (Fesus and Piacentini, 2002). Of interest is that endogenous TG2 has been shown to stimulate differentiation of cells including neurons, astrocytes and neutrophils (Campisi et al., 1992; Tucholski et al., 2001; Balajthy et al., 2006; Numinskaya and Belkin, 2012). Previous work from our group indicates that TG2 plays a prominent role in the timing of differentiation of OPCs into myelin-forming OLGs, i.e., inhibition of TG2 activity delays OPC differentiation and TG2 knock-out mice lag behind in remyelination of axons in the corpus callosum upon cuprizone-induced demyelination (van Strien et al., 2011a). TG2 immunoreactivity was also found in activated microglia and hypertrophic astrocytes in active and chronic active MS lesions (van Strien et al., 2011c; Espitia Pinzón et al., 2017a), likely induced by inflammatory mediators that are present. Astrocytes contribute to remyelination failure in MS. Activated astrocytes proliferate and form a dense network of hypertrophic cells, known as the astroglial scar, which is beneficial because it seals of inflammation in the CNS into focal areas (Williams et al., 2007). However, the scar also impedes remyelination by inhibiting migration of OPCs into the lesions (Fawcett and Asher, 1999) and maturation of OPCs within the lesions (Motavaf et al., 2017). Upon demyelination, activated astrocytes transiently secrete soluble factors, such as growth factors and cytokines (Wiese et al., 2012), and deposit extracellular matrix (ECM) proteins, including fibronectin and hyaluronan, that prevent OPC maturation (Back et al., 2005; Stoffels et al., 2013b). In MS lesions, fibronectin dimers are cross-linked to form aggregates and hyaluronan is present in its high molecular weight form, and their persistent presence contribute to the non-permissive nature of this scar for remyelination and to impaired remyelination by inhibiting OPC differentiation (Back et al., 2005; Sisková et al., 2006; Stoffels et al., 2013a). Of interest, astrocyte-derived TG2 affects fibronectin deposition, but not aggregation upon demyelination (Espitia Pinzón et al., 2017b), and TG2-positive astrocytes in MS lesions partly co-localize with fibronectin (van Strien et al., 2011c).

Based on previous observations that both astrocytes and OPCs express TG2, we questioned here whether astrocytic or oligodendroglial TG2 affects OPCs in a cell-specific manner to modulate their differentiation, and therefore the (re)myelination process. To this end, we used co-culture systems of astrocytes and OPCs, and/or neurons or myelinating spinal cord cultures with an astrocyte feeding layer that expressed human TG2 or that were treated with recombinant TG2 and studied myelin formation. Moreover, we examined TG2 immunoreactivity in human OPCs during development and in MS lesions. We also analyzed the effect of inflammatory mediators that are present in MS lesions on TG2 expression in OPCs and considered whether this affected early OPC differentiation. A better understanding of the role of either astrocytic or oligodendroglial TG2 in OPC differentiation is of therapeutic interest to overcome failing remyelination in MS.

## Materials and Methods

### TG2 Lentiviral Transduction

Human wild-type TG2 (hTG2; gift from prof. K. Mehta, University of Texas, M.D. Anderson Cancer Center, Houston, TX, United States) and GFP were cloned into the lentiviral vector pCDH (puro). As a control, “empty” plasmid, i.e., pCDH vector, was used (MOCK). For the production of the lentiviral particles, HEK 293T cells were cultured in a poly-L-lysine- (PLL, 5 μg/ml, Sigma-Aldrich, St. Louis, MO, United States) coated 6 well plate at 1.0 × 10^6^ cells per well (2 ml) in Dulbecco’s Modified Eagle Medium (DMEM, Life Technologies, Paisley, United Kingdom) supplemented with 10% fetal bovine serum (FBS; Bodinco, Alkmaar, Netherlands), L-glutamine and penicillin and streptomycin (Life Technologies, United States). Cells were transfected with the constructs, i.e., the “empty” pCDH vector or the vectors containing the human wild-type TG2 or GFP, packaging and envelop plasmids (VSV-G and CMVdR8.1) via calcium phosphate transfection or Lipofectamine^TM^ 2000 Transfection Reagent (Invitrogen, Carlsbad, CA, United States) as described in the manufacturer’s instructions. At 48 h after transfection, 2 ml of conditioned medium was collected and filtered through a PVDF membrane based filter (0.45 μm pore size) and either used immediately or stored at −80°C until further use. OPCs and astrocytes were transduced before the shake-off by exposing the cells overnight at 37°C to lentiviral particles and polybrene (8 μg/ml; Sigma-Aldrich, St. Louis, MO, United States). The cells were left to recover in DMEM supplemented with 10% FBS (Capricorn Scientific, Ebsdorfergrund, Germany), L-glutamine, penicillin and streptomycin for 48 h and selected as described below.

### Single Cell Cultures

This study was carried out in accordance with the recommendations of national and local experimental animal guidelines and regulations. The protocol was approved by the Institutional Animal Care and Use Committee of the University of Groningen (Netherlands). Primary glial cultures were generated from 1-3-day-old Wistar rats (Charles River) as previously described (Maier et al., 2005; Bsibsi et al., 2012). Briefly, after 12 days in culture on PLL-coated 75 cm^2^ cell culture flasks (Nalge Nunc, Naperville, IL, United States), OPCs and astrocytes were isolated via a shake-off procedure. Contaminating microglia were removed by shaking the flasks at 150 rpm for 1 h at 37°C on an orbital shaker (Innova 4000, New Brunswick Scientific, United States).

#### Oligodendrocytes

Subsequently, flasks were shaken at 240 rpm overnight at 37°C to detach OPCs. Floating OPCs were further purified via differential adhesion on non-tissue culture dishes (Greiner Bio-one) and cultured in PLL-coated plates in defined SATO medium (Maier et al., 2005) in the presence of platelet-derived growth factor-AA (PDGF-AA, 10 ng/ml; Peprotech; London, United Kingdom) and fibroblast growth factor-basic (FGF-2, 10 ng/ml; Peprotech) to synchronize the OPCs. Cells were plated at a density of 0.3–0.4 × 10^6^ cells per well (6 well plate, Corning, Lowell, MA, United States) for western blot analysis. For immunocytochemical studies cells were plated on 13-mm PLL-coated coverslips (VWR, Amsterdam, Netherlands) in 24 well plates (Corning) at 3.5 × 10^5^ cells per well or on PLL-coated 8-well chamber slides (Labtek, Nunc) at 2.5 × 10^4^ cells per well. After 48 h, differentiation was induced by growth factor withdrawal and cells were grown in SATO medium supplemented with 0.5% FBS. OPCs differentiate via pre-myelinating OLGs (immature OLGs; 3 days after initiating differentiation) into myelinating OLGs (mature OLGs; 7 days after initiating differentiation). Recombinant guinea pig TG2 (gpTG2, Sigma-Aldrich, 50 μg/ml), astrocyte conditioned-medium (1:1 ratio with SATO), and rat recombinant tumor necrosis factor (TNF)-α (Peprotech, 10 ng/ml), rat recombinant interleukin (IL)-1β (Peprotech, 10 ng/ml) or rat recombinant interferon (IFN)-γ (Peprotech, 500 U/ml) were added at the indicated stages of OLG development.

#### Astrocytes

Astrocytes were obtained by adding trypsin to the remaining monolayer of cells. Subsequently, the astrocytes were cultured in 162 cm^2^ flasks (Corning Costar, Lowell, MA, United States) in DMEM supplemented with heat-inactivated 10% FBS. To obtain astrocyte-conditioned medium from control and transduced cells, astrocytes were cultured in a 6 well plate (1 × 10^6^ cells per well) in DMEM supplemented with heat-inactivated 10% FBS. After 24 h, medium was replaced with 1 ml SATO medium containing 0.5% FBS. After 24 h, medium was collected and filtered through a 0.45 μm filter and stored at −20°C until further use. For spinal cord cultures, control and transduced astrocytes were plated on 13-mm PLL-coated coverslips in a 24 well plate (7.0 × 10^4^ cells per well) to form an astrocyte monolayer in 3 days. For western blot analysis, cells were plated on a 6-well plate at 0.3–0.4 × 10^6^ cells per well and collected after 48 h.

### Myelinating Co-cultures

#### Myelinating Spinal Cord Cultures

Myelinating spinal cord cultures were generated from 15-day-old Wistar rat embryo’s (Charles River) as described before (Sorensen et al., 2008), with minor modifications. Briefly, after removal of meninges, the isolated spinal cords were minced and enzymatically digested. Tissue digestion was stopped and cells were triturated and centrifuged at 1,000 rpm for 5 min. The cell pellet was resuspended in plating medium [PM: 50% DMEM (Life Technologies, United States), 25% horse serum (Invitrogen), 25% HBSS (Life Technologies, United States) and 2 mM glutamin], and cells were cultured on 13-mm coverslips in a 24-well plate with the indicated astrocyte monolayer (control or transduced) at a density of 2.0 × 10^5^ cells/coverslip (500 μl/well). After 2 h, 500 μl of differentiation medium [DM: DMEM supplemented with 1 mg/ml holotransferrin (Sigma-Aldrich, United States), 20 mM putrescine (Sigma-Aldrich, United States), 4 μM progesterone (Sigma-Aldrich, United States), 6 μM selenium (Sigma-Aldrich, United States), 10 ng/ml insulin (Sigma-Aldrich, United States)] was added. Half of the medium was replaced every second day with fresh DM. After 12 days *in vitro* (div), insulin was omitted from DM, and cultures were treated with vehicle (phosphate buffered saline, PBS) or 50 μg/ml gpTG2. gpTG2 was added upon each medium change, i.e., every 2 days. The cultures were analyzed at 26–28 div.

#### Myelinating DRGN-OPC Co-cultures

Primary rat dorsal root ganglia neurons (DRGNs) were isolated from 15-days-old Wistar rat embryo’s (Charles River), as described before (Chan et al., 2004; Stancic et al., 2012), with minor modifications. Dissociated DRGNs were plated as 40 μl drops at a density of 6 × 10^4^ cells on 13 mm coverslips (0.5 ml) that were pre-coated with PLL, followed by growth-factor-reduced matrigel (1:40 dilution; BD Bioscience). OPCs (control or transduced) were seeded onto DRGNs after 16–21 div at a 1:1 ratio in DMEM supplemented with 1% ITS supplement (Sigma-Aldrich, United States), 0.25% FBS, D+-glucose (4 mg/ml, Sigma-Aldrich, United States), L-glutamine and penicillin and streptomycin. After 2 days in co-culture, the cultures were treated with vehicle (PBS) or 50 μg/ml gpTG2. Co-cultures were maintained for up to 14 days with medium changes at every third day. GpTG2 was added upon medium changes.

### Human Material

In compliance with local and national ethical and legal guidelines, approval by an ethics committee for the use of post-mortem human material was not required. We did obtain written informed consent for brain autopsy and the use of brain tissue and clinical information for scientific research by either the donor or the next of kin.

#### MS Lesions

Post-mortem human (sub)cortical tissue was obtained from Netherlands Brain Bank (NBB, Amsterdam, Netherlands). Formalin-fixed, paraffin-embedded tissue sections containing chronic active white matter lesions or remyelinating lesions were included from 3 clinically diagnosed and neuropathologically verified MS patients (age range: 41–53 years).

#### Human Cerebellum

Human tissue was obtained from the department of Pathology at the Amsterdam UMC, VU University Amsterdam, after post-mortem examination. Formalin-fixed, paraffin-embedded tissue sections containing human cerebellum tissue at gestational week 28 till post-partum month 2 (*n* = 8) were included after (preterm) births.

### Immunocyto-and Immunohistochemical Analysis

#### OPC Monocultures

Paraformaldehyde (4% PFA, Merck) fixed cells were permeabilized with ice-cold methanol for 10 min. After a 30-min block with 4% bovine serum albumin (BSA), cells were incubated for 60 min at room temperature (RT) with primary antibodies (see Table 1), i.e., anti-myelin basic protein (MBP) (1:250, Serotec, Oxford, United Kingdom) or anti-TG2 (1:500, Ab2, Labvision, Fremont, CA, United States) diluted in 4% BSA in PBS. Next, the cells were rinsed with PBS and incubated for 25 min with appropriate TRITC-conjugated secondary antibody (1:50, diluted in 4% BSA in PBS; Jackson ImmunoResearch, Westgrove, PA, United States). Nuclei were stained with DAPI (1 μg/ml, Sigma-Aldrich, United States), and mounting medium (Dako, Heverlee, Belgium) was added to prevent image fading. The cells were analyzed with a conventional fluorescence microscope (Olympus ProVis AX70 or Leica DMI 6000 B). OLGs were characterized by morphology, i.e., cells with a typical astrocytic morphology were excluded, and in each experiment at least 250 cells were scored as either MBP-positive or MBP-negative (“differentiation”). In addition, positive cells bearing MBP-positive membranous structures spread between the cellular processes were identified as myelin membrane-forming, irrespective of the extent of membrane formation (“myelination”).

**TABLE 1:** Primary antibodies used during immunohistochemistry, immunocytochemistry and western blot analysis.

**Antibody**	**Manufacturer**	**Species**	**Dilution IHC/ICC**	**Dilution western blot**
Anti-MBP	Serotec	Rat	1:250	n.a.
Anti-TG2 (Ab1)	Neomarkers	Mouse	1:200	n.a.
Anti-TG2 (Ab2)	Labvision	Mouse	1:500	n.a.
Anti-TG2 (Ab3)	Labvision	Mouse	1:500	1:1,000
Anti-neurofilament-H	EnCor Biotechnology	Chicken	1:5,000	n.a.
Anti-Olig2	Millipore	Rabbit	1:400	n.a.
Anti-PLP	Biorad	Mouse	1:500	n.a.
Anti-MHC-II (clone LN3)	Pierce	Mouse	1:1,000	n.a.
Anti-PDGFRα	R&D systems	Goat	1:100	n.a.
Anti-actin	Sigma-Aldrich	Mouse	n.a.	1:1,000

*n.a., not applicable; IHC/ICC, immunohistochemistry/immunocytochemistry.*

#### Myelinating Cultures

Cultures were fixed at the indicated time points in 4% PFA for 15 min, washed with PBS and incubated at RT in 0.5% Triton X-100 (TX-100) in 5% normal goat serum (NGS) for 40 min. After PBS washing, the cells were incubated for 2 h at RT with primary antibodies (see Table 1), i.e., anti-MBP (1:250) and anti-neurofilament-H (1:5,000, EnCor Biotechnology Inc., Gainesville, FL, United States) diluted in 2% NGS in PBS. Staining was visualized by an incubation for 30 min at RT with appropriate Alexa-conjugated secondary antibodies diluted in 2% NGS (1:500, Invitrogen, United States). Coverslips were mounted in mounting media. All analyses were performed using a confocal laser scan microscope (Leica SP8 AOBS CLSM, Leica Microsystems, Heidelberg, Germany) equipped with Leica Confocal Software. Fluorescence images were acquired sequentially and processed using Adobe Photoshop CS3 (Adobe Systems, San Jose, CA, United States). Data were obtained from 3 independent experiments, while in each experiment 4–5 images at 20x magnifications per coverslip and 1–2 coverslips per condition were analyzed. To quantify the percentage of myelinated axons, axonal density (neurofilament staining) was first measured in ImageJ as a percentage of the total area of the image. In order to exclude immunoreactivity associated with OLG cell bodies, myelination was manually traced in Adobe Photoshop CS3. The percentage of myelinated axons was calculated as an area in pixels in each image occupied by both myelin and axons divided by the axonal density.

#### MS Brain Material

After rapid autopsy (mean postmortem delay: 7.4 h) tissue samples were fixed in 10% formalin for 30 days and embedded in paraffin. Sections (10 μm) were cut and mounted on positively charged glass slides (Menzel-Glaser SuperFrost plus, Braunschweig, Germany), and dried overnight at 37°C. Upon use, sections were heated on a hot plate for 30 min at 58°C, before they were deparaffinized in xylene-replacement (Sigma-Aldrich), and rehydrated through a series of 100, 98, 80, and 70% ethanol and distilled water. For subsequent antigen retrieval, sections were rinsed in 0.01 M citrate buffer (pH 6.0) and subsequently heated in a steam cooker for 30 min in the same buffer. After antigen retrieval, the sections were allowed to regain RT, rinsed in Tris-buffered saline (TBS), and incubated for 20 min in TBS containing 0.3% hydrogen peroxidase and 0.1% sodiumazide. Non-specific binding sites were blocked with 3% BSA in TBS-T (blocking solution) for 30 min at RT. Subsequently, sections were incubated overnight at 4°C with primary antibodies (see Table 1), i.e., mouse anti-TG2 (ab1, 1:200, Neomarkers) and rabbit anti-Olig2 (1:400, Millipore), diluted in blocking solution. The next day, sections were washed in TBS and incubated for 30 min at RT in Alkaline Phosphatase (AP) labeled rabbit IgG Polymer Reagent (ImmPRESS^TM^, Vector Laboratories, Burlingame, CA, United States) washed in TBS and incubated for 30 min at RT in horseradish peroxidase (HRP) labeled mouse IgG Polymer Reagent (Envision, DAKO). Sections were washed again in TBS and incubated for 20 min in Liquid Permanent Red (LPR, DAKO) as a chromogen to stain for Olig2. After several washes in TBS, sections were incubated for 20 min with Vector SG (3 drops in 5 ml PBS with 3 drops of hydrogen peroxide, Vector Laboratories, Burlingame, CA, United States) as a chromogen to stain for TG2. Sections were washed in Tris–HCL and running water and were allowed to dry for 1 h at 37°C. Sections were cleared in xylene and coverslipped in Entellan (Merck, Darmstadt, Germany).

In addition, the lesions present in MS tissue were defined by the loss of myelin as observed by proteolipid protein (PLP), a marker of myelin, and the immunological activity status by major histocompatibility complex (MHC) class II staining. Upon use, sections were heated on a hot plate for 30 min at 56°C, before they were deparaffinized in xylene-replacement (Sigma-Aldrich, United States), and rehydrated through a series of 100, 96, 90, 80, and 70% ethanol and distilled water. For subsequent antigen retrieval, sections were rinsed in Tris–EDTA buffer (pH 9.0) and subsequently heated in a steam cooker for 30 min in the same buffer. After antigen retrieval, the sections were allowed to regain RT, rinsed in TBS, and incubated for 15 min in TBS containing 1% hydrogen peroxidase and 0.1% sodiumazide. Non-specific binding sites were blocked with 5% non-fat dried milk (Campina) in TBS-T for 20 min at RT. Subsequently, sections were incubated overnight at 4°C with primary antibodies (see Table 1), i.e., anti-PLP (1:500, Biorad) or anti-MHC-II (1:1,000, clone LN3, Pierce), diluted in blocking solution to, respectively, identify myelin staining and MHC-II positive monocytes/macrophages or microglia. The next day, sections were washed in TBS and incubated for 2 h at RT with corresponding biotinylated IgG’s (1:400; Jackson ImmunoResearch) in TBS-T. Sections were washed in TBS and incubated with HRP-labeled avidin-biotin complex (ABC complex, 1:400, Vector Laboratories, Burlingame, CA, United States) in TBS-T for 1 h at RT. Sections were washed with TBS and Tris–HCl. Peroxidase activity was visualized using 3,3-diaminobenzidine (DAB, Sigma-Aldrich, St. Louis, MO, United States) as a chromogen. Sections were washed with Tris–HCl running tap water. Finally, sections were counterstained with haematoxylin and washed in running tap water. After dehydration in graded ethanol solutions, sections were cleared in xylene and coverslipped in Entellan. All sections were examined on a Leica DM5000B microscope equipped with a Nuance Multispectral Imaging System (Perkin Elmer Inc., MA, United States). Negative controls were performed by omitting the primary antibody resulting in no immunohistochemical signal (data not shown).

#### Human Cerebellum

Formalin-fixed paraffin embedded tissue sections (6 μm) of human cerebellum obtained at gestational week 28 till post-partum month 2 (*n* = 8) were used to determine the presence of TG2 in OPCs. The sections were deparaffinized, rehydrated, and pre-treated with citrate buffer (pH 6.0) for antigen retrieval. Subsequently, the sections were co-incubated overnight at 4°C with primary antibodies (see Table 1), i.e., anti-TG2 (1:500, Ab3, Labvision) and anti-platelet-derived growth factor receptor (PDGFR)α (1:100, R&D systems, Abingdon, United Kingdom). After rinsing the sections with TBS, the sections were co-incubated with appropriate fluorescently labeled secondary antibody to detect PDGFRα (1:400, Invitrogen, United States) and TG2 (1:400, Invitrogen) immunoreactivity. Sections were embedded in Vectashield mounting medium (Vector Laboratories, Burlingame, CA, United States) and examined on a Leica confocal laser scanning microscope (Leica, Rijswijk, Netherlands).

### Western Blot Analysis

Cells were washed several times with PBS (pH 7.4), harvested by scraping, and centrifuged for 7 min at 7,000 rpm at RT. Pellets were lysed in TNE lysis buffer (50 mM Tris–HCl, 5 mM EDTA, 150 mM NaCl, 1% Triton X-100, and protease inhibitor cocktail; Complete Mini; Roche Diagnostics GmbH), pH 7.4). Protein concentrations were determined in a Bio-Rad DC protein assay (Bio-Rad, Hercules, CA, United States) using BSA as a standard. Equal amounts of proteins (20 μg for astrocytes and 30 μg for OLGs) were diluted with reducing sample buffer and heated for 5 min at 98°C. Proteins were separated by 10% SDS-PAGE and transferred to an Immobilon-FL transfer membrane (Millipore, Bedford, MA, United States) using a wet blotting system (Biorad) and glycine-Tris-methanol buffer. The membranes were rinsed with PBS and incubated for 1 h in blocking solution (Li-Cor Biosciences, Lincoln, NE, United States). After washing with PBS containing 0.1% Tween-20, the membranes were incubated overnight with primary antibodies (see Table 1; TG2, 1:1,000, Ab3, Labvision; actin, 1:1,000, Sigma-Aldrich, United States) diluted in 50% blocking solution in PBS containing 0.1% Tween-20. After washing with PBS containing 0.1% Tween-20, the membranes were incubated for 1 h with appropriate IRDye-conjugated antibodies (1:5,000, Li-Cor Biosciences; IRDye) and washed with PBS containing 0.1% Tween-20. The signals were detected using the Odyssey Infrared Imaging System and software (Li-Cor Biosciences, United States) and analyzed in Scion image software (Scion Corporation, Maryland, United States).

### Statistical Analysis

Cell data were expressed relative to vehicle-treated (PBS or MOCK-transduced) control cells, which were set to 100% in each independent experiment. Statistical analysis was performed in IBM SPSS software, version 20.0 (IBM Corp, NY, United States). The Shapiro-Wilk procedure was first applied to test for a normal distribution of the data. When normally distributed, the one sample *t*-test was used when a single treatment was compared to a control group. Multiple group comparisons of data were performed using one-way ANOVA followed by Dunnett’s *post hoc* test for multiple comparisons compared to the control groups. Alternatively, when the data were not normally distributed (for DRGN-OPC co-cultures after lentiviral upregulation of TG2 in OPCs), a Kruskal–Wallis test was performed followed by a Mann–Whitney U *post hoc* analysis. Holm’s sequential Bonferroni correction was used to account for multiple testing during non-parametric testing. Data represent means of at least three independent experiments + standard error of the mean (SEM). Statistical differences were indicated with ^*^P < 0.05, ^∗∗^*P* < 0.01 or ^∗∗∗^*P* < 0.001.

## Results

### Human TG2 Expression in Astrocytes Does Not Significantly Affect OPC Differentiation and Myelin Membrane Formation

**FIGURE 1:**
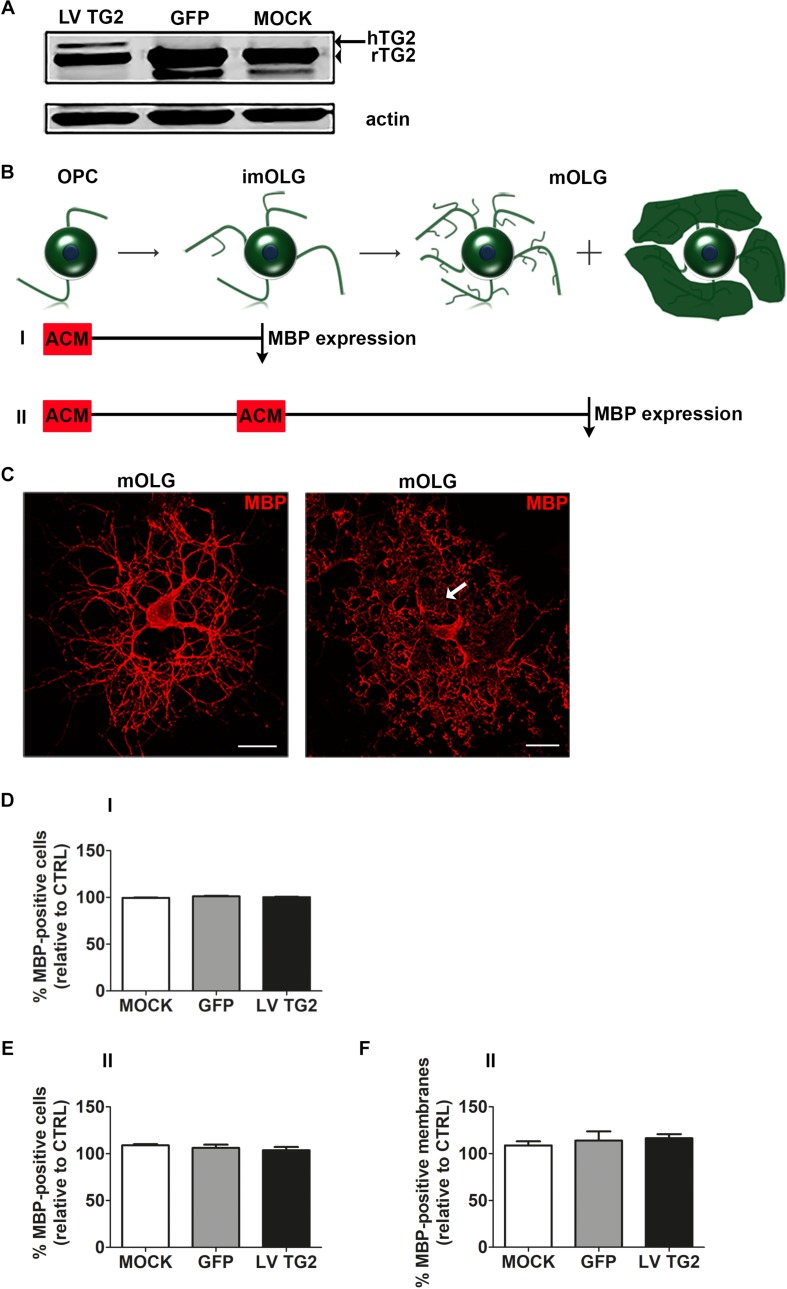
Astrocyte-conditioned medium from human TG2 expressing cells does not significantly affect OPC maturation. **(A)** Wild-type human TG2 (LV TG2), GFP and empty vector (MOCK) were lentivirally expressed in astrocytes and subjected to western blot analyses. Note that lentiviral expression of wild type human TG2 (LV TG2) in primary rat astrocytes results in an additional band indicative of the human TG2 (hTG2, arrow), which has a slightly higher molecular weight than endogenous rat TG2 (rTG2; 78 kDa, arrowhead). **(B–F)** Schematic representation of the experimental set up is shown in **B**. Astrocyte conditioned medium (ACM) derived from lentivirally transduced astrocytes (MOCK, GFP or LV TG2) is directly added to oligodendrocyte progenitor cells (OPCs, monocultures) and analyzed at the immature oligodendrocyte stage (imOLG, “I,” **D**) or added both at the OPC stage and the imOLG stage and analyzed at the mature OLG stage (mOLG, “II,” **E,F**). OPC differentiation **(D,E)** and myelin membrane formation **(F)** are, respectively, assessed as the percentage of myelin basic protein (MBP)-positive cells and the percentage of myelin membrane bearing MBP-positive cells. Representative images of MBP (myelin marker, red)-positive cells without (left) and with myelin membranes (right, arrow) are shown in **C**. Scale bar is 100 μm. Data are shown as mean + SEM, calculated as average percentage compared to OPCs that received non-conditioned medium (CTRL), which was set to 100% in each independent experiment (*n* = 4). The percentage of MBP-positive cells in control cells is 1.6 ± 0.2% at the imOLG stage **(D)** and 16.7 ± 0.2% at the mOLG stage **(E)**, the percentage of myelin membranes at the mOLG stage is 35.6 ± 18.0% **(F)**. Statistical analyses were performed using a one-sample *t*-test (not significant).

To examine whether the observed delay in remyelination in TG2 –/– mice (van Strien et al., 2011a) is an indirect effect of astrocytes on OPC differentiation, the effect on OPC differentiation of conditioned medium derived from astrocytes expressing wild-type hTG2 was examined. First, we showed that MOCK- and GFP-transduced cells only express endogenous 78 kDa rat TG2, whereas in hTG2-transduced astrocytes hTG2 appeared at a slightly higher molecular weight than endogenous rat TG2 in TG2-transduced cells (Figure 1A, indicated by an arrow and an arrowhead, respectively, see Supplementary Figure 1 for full blot). The percentage of hTG2 in TG2-transduced cells relative to endogenous TG2 was 22.0% ± 0.7%. Remarkably, total TG2 expression levels were similar between hTG2- and GFP- and MOCK-transduced astrocytes, indicating that astrocytes compensated for hTG2 expression by reducing endogenous TG2 levels. Notably, in MOCK- and GFP-transduced astrocytes, but not hTG2-transduced cells, an additional TG2-reactive band at approximately 75 kDa was visible. This has been shown by others as well (Kumar and Mehta, 2012; Boroughs et al., 2014) and may represent endogenous TG2 protein that is truncated at the 3′ end (Antonyak et al., 2006).

As astrocytes affect OPC maturation indirectly through astrocyte-derived secreted factors, which may include secreted TG2 (Adamczyk et al., 2015), the effect of conditioned medium of virally transduced astrocytes (ACM), i.e., MOCK-ACM, GFP-ACM or that express hTG2 (TG2-ACM), on OPC differentiation was examined. Differentiation of OPCs involves their progression through different developmental stages from immature, pre-myelinating OLGs (Figure 1B, imOLGs, 3 days after initiating differentiation) toward mature, myelinating OLGs (Figure 1B, mOLG, 7 days after initiating differentiation). To establish whether OPCs were differentiated, cells were stained for MBP, a major myelin specific protein that is imperative for myelination (Boggs, 2006), and scored as either MBP-positive or MBP-negative (Figure 1C shows MBP-positive cells without (left) and with myelin membranes (right)). Exposure to MOCK-ACM, GFP-ACM, or TG2-ACM at the OPC stage (experimental set up “I” in Figure 1B) did not significantly affect the relative percentage of MBP-expressing cells at the immature stage compared to exposure to non-conditioned medium (Figure 1D). Similarly, continuous presence of MOCK-ACM, GFP-ACM or TG2-ACM upon differentiation till the mature OLG stage (experimental set up “II” in Figure 1B) showed no apparent effect on differentiation (Figure 1E). The MBP-positive cells bearing membranous structures spread between the cellular processes (Figure 1C, arrow) reflect the ability of OLGs to form myelin membranes. Continuous exposure of TG2-ACM upon differentiation toward mature OLGs (experimental set up “II” in Figure 1B) hardly, if at all, affected myelin membrane formation of mature OLGs compared to non-conditioned medium, MOCK- or GFP-ACM (Figure 1F), indicating that hTG2-expressing astrocytes do not modulate OPC differentiation via secreted factors.

**FIGURE 2:**
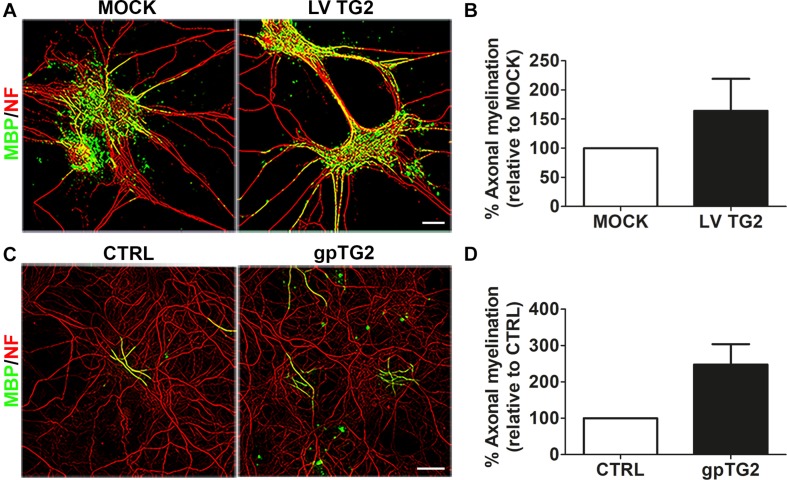
Astrocyte-derived human TG2 has no significant effect on myelination in spinal cord cultures. **(A,B)** Wild-type human TG2 (hTG2) and empty vector were lentivirally expressed in astrocytes (LV TG2 and MOCK, respectively) which were used as an astrocyte feeding layer in myelinating spinal cord cultures. Myelination was measured as percentage of myelinated axons, visualized with myelin basic protein (MBP, myelin marker, green) of total neurofilament (NF, red)-positive axons at 26–28 days *in vitro*. Representative images are shown in **A**. The relative percentage of myelinated axons in spinal cord cultures with hTG2-expressing astrocytes to MOCK treated astrocytes is shown in **B** (*n* = 3). **(C)** Spinal cord cultures on a control astrocyte feeding layer were treated with vehicle (PBS) or with recombinant guinea pig TG2 (gpTG2, 50 μg/ml every 2 days from day 12 *in vitro* for 14–16 days). Representative images are shown in **C**. The relative percentage of myelinated axons in gpTG2 treated spinal cord cultures compared to vehicle-treated (CTRL) cultures is shown in **D** (*n* = 4). Data are shown as mean + SEM, calculated as average percentage compared to MOCK-transduced **(B)** or vehicle-treated astrocytes (**D**, CTRL), which were set to 100% in each independent experiment. Statistical analyses were performed using a one-sample *t*-test (not significant). Scale bar is 100 μm.

To examine whether hTG2-expressing astrocytes may affect OPC maturation via signals other than secreted signals, hTG2-expressing astrocytes and MOCK-transduced astrocytes were used as a feeding layer in an *in vitro* myelinating spinal cord culture (Sorensen et al., 2008). Subsequent analysis of the percentage of myelinated axons, as assessed with a double labeling of MBP and the axonal marker neurofilament-H (NF; Figure 2A), revealed that hTG2 expression in astrocytes slightly, but not significantly, enhanced the percentage of myelinated axons by approximately 60% (Figure 2B; *p* = 0.365). Western blot analysis showed that hTG2 is hardly detected in hTG2-expressing astrocyte conditioned medium (ACM) (data not shown). Therefore, to exclude the possibility that astrocyte secreted TG2 levels were simply too low or were taken up by other cells in these myelinating culture set-up, cells were also treated with recombinant gpTG2. Continuous exposure of exogenous gpTG2 to spinal cord cultures with a control astrocyte feeding layer at the time of OPC differentiation, i.e., from 12 div onward, increased axonal myelination (Figure 2C) by approximately 250%. This result, however, did not reach significance due to variability between the different experiments (Figure 2D; *p* = 0.078). Hence, hTG2-rexpressing astrocytes and exogenously added gpTG2 do not significantly affect the percentage of myelinated axons in an *in vitro* myelinating culture on an astrocyte feeding layer, indicating that astrocyte-derived TG2 is not likely to affect OPC maturation. However, since we could not exclude direct developmental stage-dependent effects of TG2 on OPC differentiation/maturation, we next examined the effect of recombinant gpTG2 treatment on different stages of OLG development in monocultures.

### Addition of TG2 Has a Distinct Developmental Stage-Dependent Effect on OPC Maturation in OLG Monocultures

**FIGURE 3:**
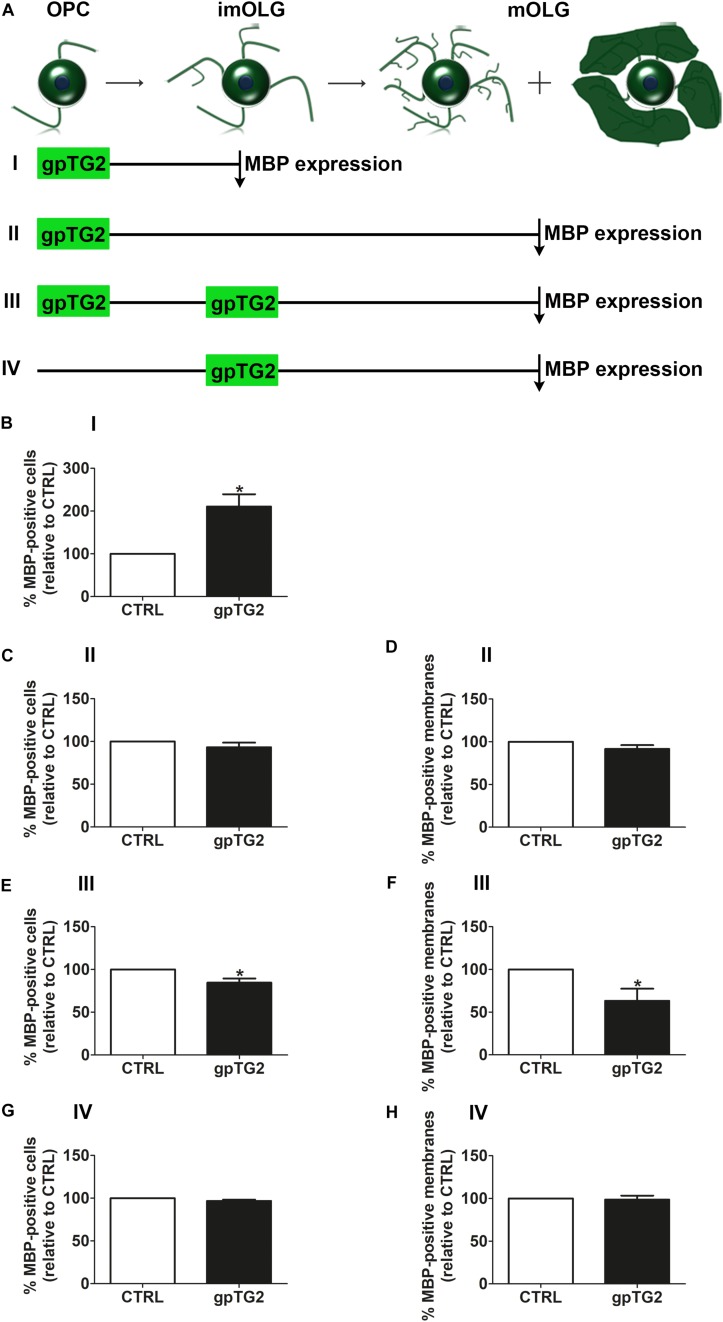
Addition of TG2 has a developmental stage-dependent effect on OPC maturation. Exogenous guinea pig TG2 (gpTG2) was added to different developmental stages of the oligodendrocyte (OLG) lineage. Schematic representation of the experimental set up is shown in **A**. gpTG2 was added once to oligodendrocyte progenitor cells (OPCs) and analyzed at the immature OLG (imOLG, “I,” **B**, *n* = 5) and mature OLG stage (mOLG, “II,” **C,D**, *n* = 5), added both at the OPC stage and the imOLG stage and analyzed at the mOLG stage (“III,” **E,F**, *n* = 8), or added at the imOLGs and analyzed at the mOLG stage (“IV,” **G,H**, *n* = 5). OPC differentiation **(B,C,E,G)** and myelin membrane formation **(D,F,H)** are, respectively, assessed as the percentage of myelin basic protein (MBP)-positive cells and the percentage of myelin membrane bearing MBP-positive cells. Data are shown as mean + SEM, calculated as average percentage compared to vehicle-treated (CTRL), which was set to 100% in each independent experiment. The percentage of MBP-positive cells in vehicle-treated cells is 6.3 ± 1.6% at the imOLG stage **(B)** and 20.8 ± 11.3% at the mOLG stage **(C,E,G)**, the percentage of myelin membranes at the mOLG stage is 40.8 ± 24.4% **(D,F,H)**. Statistical analyses were performed using a one-sample *t*-test (^*^p < 0.05).

As the timing of OPC differentiation is important for remyelination, the effect of exogenous addition of recombinant gpTG2 on early OPC differentiation was first examined. A single addition of gpTG2 to OPCs and analyses of the percentage of MBP-positive cells at the immature OLG stage (experimental set up “I” in Figure 3A) significantly increased the percentage of MBP-positive cells by 2-fold (Figure 3B; *p* < 0.05). This increase in OPC differentiation was no longer apparent at the mature OLG stage (experimental set up “II” in Figures 3A,C) indicating that a transient exposure to gpTG2 at the OPC stage accelerated differentiation. In addition, gpTG2, when added once at the OPC stage did not affect myelin membrane formation (Figure 3D). In contrast, however, when gpTG2 was continuously present during differentiation, i.e., added to OPCs and re-added to immature OLGs and analyzed at the mature OLG stage (experimental set up “III” in Figure 3A), a decrease in MBP-positive cells (Figure 3E; *p* < 0.05) and myelin membrane formation (Figure 3F; *p* < 0.05) was apparent. This decrease in OPC maturation is likely not due to an effect of gpTG2 on immature OLGs, as the percentage of MBP-positive cells and myelin membranes is hardly, if at all, affected when gpTG2 was only added to immature OLGs and analyzed at the mature OLG stage (experimental set up “IV” in Figures 3A,G,H). Hence, exogenous addition of gpTG2 revealed distinct differentiation stage-dependent effects on OPC differentiation. More specifically, transient exposure of gpTG2 to OPCs accelerated differentiation at the immature OLG stage, which was lost at the mature OLG stage, while continuous exposure to gpTG2 from the OPC to mature OLG stage, decreased OPC maturation.

### Addition of TG2 to DRGN-OPC Co-cultures Increases Myelination

**FIGURE 4:**
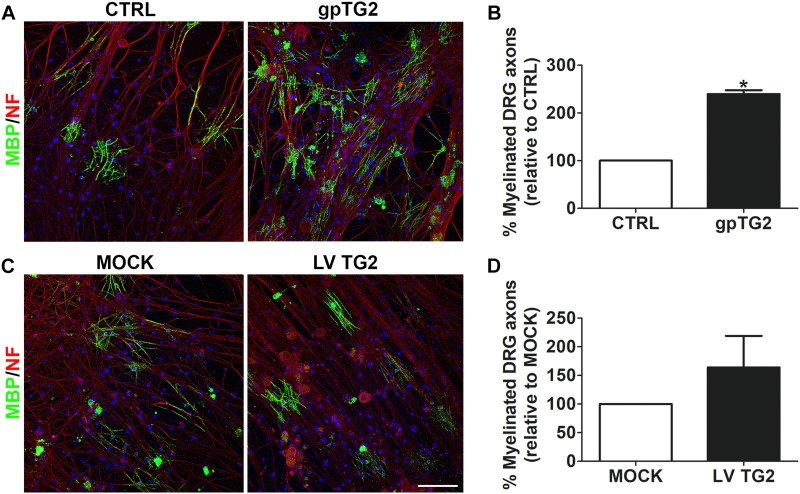
Addition of TG2 to DRGN-OPC co-cultures increases myelination. **(A,B)** Exogenous guinea pig TG2 (gpTG2, 25 μg/ml every 3 days from day 2 *in vitro* for 11 days) was added to dorsal root ganglion neurons-oligodendrocyte progenitor cell (DRGN-OPC) co-cultures. Myelination was measured as percentage of myelinated axons, visualized with myelin basic protein (MBP, myelin marker, green) of total neurofilament (NF, red)-positive axons 14 days in co-culture. Nuclei are visualized with DAPI (blue). Representative images are shown in **A**, and the relative percentage of myelinated axons in gpTG2-treated DRGN-OPC co-cultures to vehicle-treated (CTRL) co-cultures is shown in **B** (*n* = 3). **(C,D)** Wild-type human TG2 (hTG2) was lentivirally (LV TG2) overexpressed in OPCs, and plated onto DRGNs. The extent of myelination was analyzed at 14 days in co-culture. Representative images are shown in **C**, and the relative percentage of myelinated axons in DRGN-OPC co-cultures with hTG2-expressing OPCs to MOCK OPCs is shown in **D** (*n* = 3). Data are shown as mean + SEM, calculated as average percentage compared to vehicle-treated (**B**, CTRL) or MOCK-transduced OPCs **(D)**, which were set to 100% in each independent experiment. Statistical analyses were performed using a one-sample *t*-test (^*^p < 0.05). Scale bar is 100 μm.

As exogenously added gpTG2 affected OPC differentiation, we subsequently examined whether exposure to TG2 affects axonal myelination. OPC-DRGN co-cultures were continuously exposed to exogenous recombinant gpTG2 2 days after OPCs were added to DRGNs. At 14 days in co-culture, the percentage of myelinated axons was significantly increased upon addition of gpTG2 compared to vehicle-treated co-cultures (Figures 4A,B; *p* < 0.05). Remarkably, the number of DAPI-stained cells present on the DRGNs was increased in gpTG2-treated compared to control cultures, indicating that gpTG2 may also affect proliferation and/or survival. To examine whether persistent expression of endogenous TG2 in cells of the OLG lineage affects axonal myelination, OPCs were lentivirally transduced with human wild-type TG2 (LV TG2) or empty vector (MOCK) and subsequently plated onto DRGNs. At 14 days in co-culture, a similar percentage of myelinated axons of DRGNs in co-culture with TG2-overexpressing and MOCK-transduced OPC were observed (Figures 4C,D), indicating that persistent expression of TG2 in cells of the OLG lineage does not result in enhanced myelination of axons at the end of myelination. To assess whether persistent endogenous expression of TG2, similar to addition of exogenous gpTG2, may only accelerate OPC differentiation, the effect of lentivirally expression of hTG2 on OPC maturation was examined next in OLG monocultures.

### Sustained TG2 Overexpression in OLG Lineage Cells by Lentiviral Transduction Accelerates (Early) Differentiation

**FIGURE 5:**
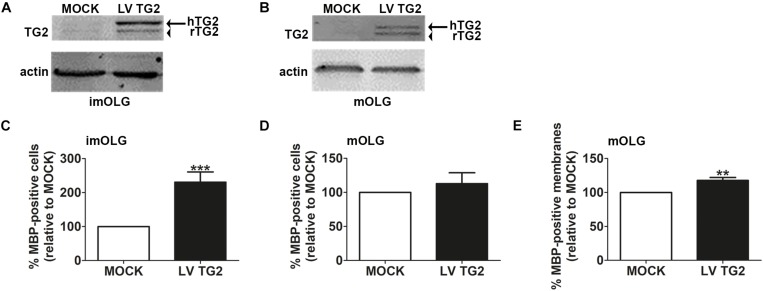
Sustained TG2 expression accelerates (early) OPC differentiation and increases myelin membrane formation. Wild-type human TG2 (LV TG2) and empty vector (MOCK) were lentivirally expressed in oligodendrocyte progenitor cells (OPCs) and subjected to western blot analyses **(A,B)** or myelin basic protein (MBP) immunocytochemistry **(C–E)** at the immature **(A,C)** and mature **(B,D,E)** oligodendrocyte (imOLG and mOLG, respectively) stage. Note that lentiviral expression of wild type human TG2 (LV TG2) in OPCs results in sustained expression of TG2 (**A,B**, arrow) and rat TG2 (**A,B**, arrowhead) upon differentiation. Actin serves as a loading control **(A,B)**. OPC differentiation **(C,D)** and myelin membrane formation **(E)** are, respectively, assessed as the percentage of MBP-positive cells and the percentage of myelin membrane bearing MBP-positive cells. Data are shown as mean + SEM, calculated as average percentage compared to MOCK-transduced OPCs, which was set to 100% in each independent experiment (**C**, *n* = 8, **D,E**, *n* = 5). The percentage of MBP-positive cells in MOCK-transduced cells is 8.7 ± 10.3% at the imOLG stage **(C)** and 24.9.7 ± 9.9% at the mOLG stage **(D)**, the percentage of myelin membranes at the mOLG stage is 38.9 ± 25.7.0% **(E)**. Statistical analyses were performed using a one-sample *t*-test (^∗∗^*p* < 0.01 and ^∗∗∗^*p* < 0.001).

We have previously shown that OPCs express TG2, while TG2 is almost not expressed in immature and mature OLGs (van Strien et al., 2011a). To assess whether persistent expression of TG2 in cells of the OLG lineage affects OPC differentiation, OPCs were lentivirally transduced with wild-type human TG2 (LVTG2) or empty vector (MOCK) and allowed to differentiate. Persistent expression of hTG2 in immature and mature OLGs was confirmed by western blotting (Figures 5A,B, respectively, see Supplementary Figure 2 for full blots). Consistent with previous observations (van Strien et al., 2011a), MOCK-transduced immature and mature OLGs showed hardly any endogenous rat TG2 band at 78 kDa, whereas hTG2-transduced cells expressed human TG2 (Figures 5A,B, arrow). Strikingly, upon expression of hTG2, also the expression of endogenous rat TG2 appeared and remained during OPC differentiation (Figures 5A,B, arrowhead). Moreover, hTG2 overexpressing OPCs significantly increased the percentage of MBP-positive cells when analyzed at the immature OLG stage (Figure 5C; *p* < 0.001), whereas the percentage of MBP-positive cells at the mature OLG stage was not significantly affected (*p* = 0.608; Figure 5D). Of note, the total number of DAPI-stained cells appeared similar at all conditions. Myelin membrane formation, however, was slightly, but significantly increased at the mature OLG stage in hTG2-expressing cells compared to MOCK-transduced cells (Figure 5E; *p* < 0.01). Thus, our findings revealed that persistent expression of TG2 in cells of the OLG lineage accelerated early OPC differentiation. As TG2 is beneficial for early OPC differentiation, we next examined whether TG2 is present in cells of the OLG lineage in MS lesions.

### TG2 Is Not Present in OPCs in MS Lesions, but Is Found in OPCs During Human Cerebellar Development

**FIGURE 6:**
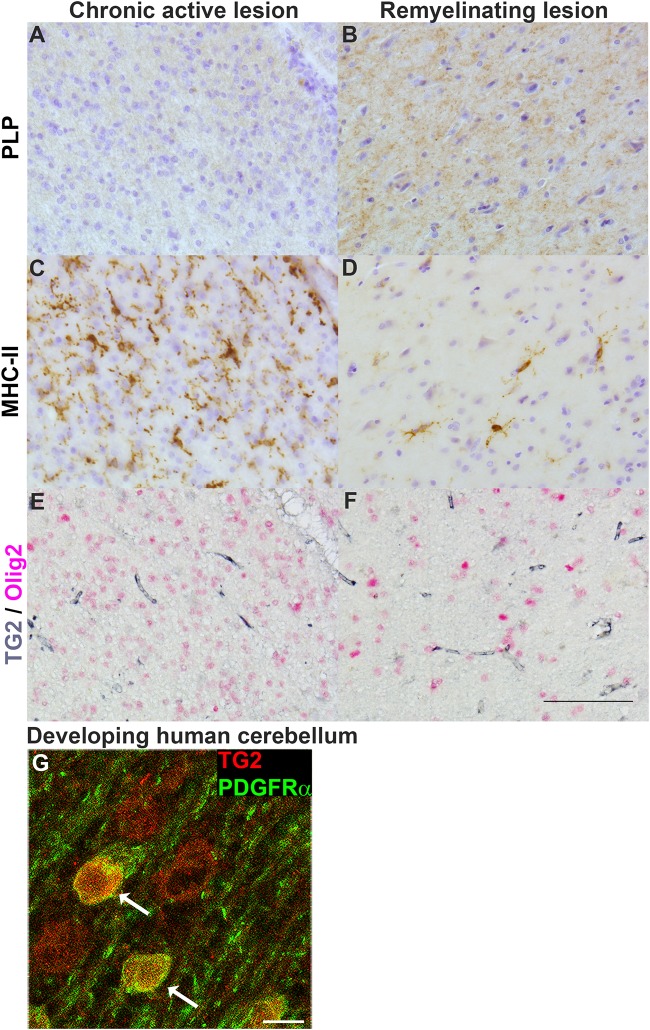
TG2 immunoreactivity is not present in OPCs in MS lesions, but is found in OPCs during human cerebellar development. Immunohistochemical stainings were performed for the myelin marker proteolipid protein (PLP) and for MHC-II positive monocytes/macrophages or microglia in chronic active lesions (**A**,**C**, respectively) and in remyelinating lesions (**B**,**D**, respectively). Immunohistochemical double labeling of TG2 (gray) and Olig2 (pink) was performed in chronic active lesions **(E)** and remyelinating MS lesions **(F)** (*n* = 3). Cell nuclei were stained by haematoxylin (blue) in PLP and MHC-II labeled sections **(A–D)**. Scale bar: 100 μm. Immunofluorescent double labeling of TG2 (red) and the OPC marker platelet-derived growth factor receptor (PDGFR)α (green) (**G**, arrows) in white matter of developing human cerebellum at gestational week 28. Scale bar: 10 μm.

To determine whether cells of the OLG lineage contain TG2 in MS lesions, a double labeling of TG2 with the OLG lineage marker Olig2 was performed in chronic active and remyelinating MS lesions. Loss of PLP immunoreactivity confirmed ongoing demyelination at the edge of chronic active lesions (Figure 6A), while small PLP-positive myelin fibers likely reflected remyelination in lesions (Figure 6B). The abundant presence of MHC-II-positive microglia in chronic active lesions was evident (Figure 6C) which was less pronounced in remyelinating lesions (Figure 6D). TG2 immunoreactivity was present in blood vessels which is a well known localization (van Strien et al., 2011c, 2015; Chrobok et al., 2019), but was virtually absent from Olig2-positive OLG lineage cells in chronic active (Figure 6E) and in remyelinating MS lesions (Figure 6F). To examine whether TG2 is expressed in differentiating human OPCs, we studied TG2 immunoreactivity in OPCs during developmental myelination of the human brain. Interestingly, TG2 immunoreactivity was observed in platelet-derived growth factor receptor alpha (PDGFRα)-positive OPCs (indicated by arrows in Figure 6G) in the developing human cerebellum, at gestational week 28. Hence, while OPCs in MS lesions do not show TG2 immunoreactivity, TG2 is present in OPCs during human cerebellar development, indicating that at the onset of normal OPC differentiation, TG2 expression is likely induced by local cues. Given that Transglutaminase expression and activity is commonly detected both in diseased tissues with inflammation and cells with inflammatory stress (Kuncio et al., 1998; Johnson et al., 2001; Kim, 2006), and TG2 expression is regulated by inflammatory mediators (van Strien et al., 2011b; Espitia Pinzón et al., 2017a), we next examined the effect of inflammatory mediators that are present in MS lesions on TG2 expression in OPCs and immature OLGs.

### IFN-γ Increased and Sustained TG2 Expression in OLG Lineage Cells, Which Did Not Correlate With Enhanced Differentiation

**FIGURE 7:**
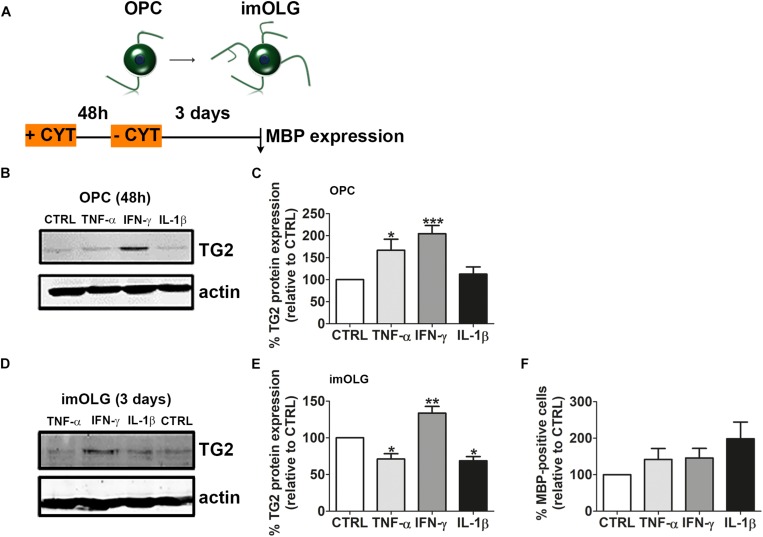
IFN-γ increases and sustains TG2 expression in OLG lineage cells, which does not correlate with enhanced differentiation. Schematic representation of the experimental set up is shown in **A**. Oligodendrocyte progenitor cells (OPCs) were left untreated (CTRL) or treated for 48 h with IFN-γ, TNF-α or IL-1β and subjected to western blot analyses **(B,C)** or allowed to differentiate for 3 days to immature oligodendrocytes (imOLG) in the absence **(A,D)** of these cytokines and subjected to western blot analyses **(D,E)** or myelin basic protein (MBP) immunocytochemistry **(F)**. Representative blots are shown in **B,D**, quantification in **C,E**. Western blot data are quantified by normalizing the optical densities of TG2 against actin, which serves as loading control. OPC differentiation **(F)** is assessed as the percentage of MBP-positive cells. Data are shown as mean + SEM, calculated as average percentage compared to control OPCs, which was set to 100% in each independent experiment (**C**, *n* = 7, **E**, *n* = 4, **F**, *n* = 4). The percentage of MBP-positive cells at the imOLG stage in control cells is 20.7 ± 15.4% in **F**. Statistical analyses were performed using a one-sample *t*-test (^*^p < 0.05, ^∗∗^*p* < 0.01 and ^∗∗∗^*p* < 0.001).

As the pro-inflammatory cytokines, TNF-α, IFN-γ, and IL-1β are present in MS lesions (Codarri et al., 2010; Mendiola and Cardona, 2018) and influence OPC maturation (Xie et al., 2016; Valentin-Torres et al., 2018) and therefore remyelination, we next analyzed whether these cytokines may modulate TG2 expression in OPCs. OPCs were therefore exposed to these cytokines for 48 h and allowed to differentiate for 3 days in the absence of cytokines (Figure 7A). Western blot analyses revealed that 48 h-exposure to IFN-γ, and to a lesser extent to TNF-α, but not to IL-1β, increased TG2 expression (Figures 7B,C; TNF-α, *p* < 0.05; IFN- γ, *p* = 0.001, for full blot of Figure 7B see Supplementary Figure 3A). Interestingly, the enhanced TG2 expression in cytokine-treated OPCs was sustained in IFN-γ-treated OPCs, i.e., the increased TG2 expression was still apparent in immature OLGs that were differentiated in the absence of cytokines (Figures 7A,D,E; *p* < 0.01, for full blot of Figure 7D see Supplementary Figure 3B), whereas TG2 expression decreased in TNF-α- or IL-1β-treated OPCs upon differentiation (Figures 7A,D,E; *p* < 0.05 for both conditions). To reveal whether the cytokine-enhanced increase in TG2 expression affects OPC differentiation, cytokine-exposed OPCs were left to differentiate without the presence of cytokines, since prolonged cytokine treatment can lead to cytotoxicity in OLGs (Robbins et al., 1987; Vartanian et al., 1995). Upon differentiation toward the immature OLG stage, the percentage of MBP-positive cells was slightly, but non-significantly, increased in TNF-α-, IFN-γ-, and IL-1β-exposed OPCs (Figure 7F). Hence, our results revealed that the for MS relevant inflammatory mediators IFN-γ, and to a lesser extent TNF-α, increased TG2 expression in OPCs, though no apparent correlation between cytokine-induced TG2 expression and increased OPC differentiation at the immature OLG stage exists, as was observed upon sustained expression of hTG2 in OLGs (Figure 5). Therefore, enhanced TG2 expression induced by inflammatory mediators is not sufficient to accelerate early OPC differentiation.

## Discussion

In previous work, we demonstrated that TG2 expression in OPCs is downregulated upon differentiation and that, in the absence of TG2, remyelination is delayed upon cuprizone-induced demyelination (van Strien et al., 2011a), which led to our hypothesis that TG2 promotes OPC maturation. Our present findings indicate that early OPC maturation is accelerated by both endogenous and exogenously supplied TG2, while factors derived from hTG2-expressing astrocytes do not significantly affect OPC maturation and myelination. Moreover, we found TG2 immunoreactivity in human OPCs during development, but not in cells of the OLG lineage that are present in chronic active and remyelinating MS lesions. Interestingly, TG2 expression in OPCs was enhanced upon exposure to IFN-γ, and sustained in immature OLGs, but did not correlate with an increase in OPC maturation. Thus, while TG2 expression and exposure promotes early OPC differentiation under physiological developmental circumstances, IFN-γ, although enhancing TG2 expression, may interfere with (accelerated) OPC differentiation in a TG2-independent manner. In addition, the absence of TG2 in cells of the OLG lineage in MS lesions may contribute to their quiescence, and therefore development of means to induce TG2 expression in local OPCs may be of therapeutic interest.

Astrocytes, when activated upon demyelination of the CNS, transiently secrete soluble factors, such as growth factors and cytokines (Wiese et al., 2012) and deposit ECM proteins, such as fibronectin and hyaluronan, that prevent OPC maturation (Back et al., 2005; Stoffels et al., 2013b). We have previously shown that astrocytes, present in demyelinating MS lesions, do express TG2 and can interact with ECM proteins, including fibronectin (van Strien et al., 2011b,c; Espitia Pinzón et al., 2017a,b). In addition, we found extracellular astrocyte-associated TG2, i.e., in extracellular deposits and on the cell-surface of astrocytes, even under control conditions (van Strien et al., 2011b; Espitia Pinzón et al., 2017a). In the present study we, therefore, questioned whether astrocyte-derived TG2 may have an effect on OPC maturation. We observed that secreted factors derived from hTG2-expressing astrocytes, as well as direct contact with hTG2-expressing astrocytes did not lead to a significant alteration in OPC differentiation or myelination of axons. However, the levels of total TG2 in hTG2-expressing astrocytes do not increase endogenous TG2 levels and hTG2 was not released at detectable levels and therefore no effect was found. Of note, in contrast to astrocytes in the cuprizone model (Skripuletz et al., 2013; Tameh et al., 2013) and in MS lesions, (Williams et al., 2007; Gudi et al., 2014; Lassmann, 2014), the hTG2-expressing astrocytes used in the present study were not subjected to an inflammatory environment which could have implications for their function. Previous work revealed that upon exposure to inflammatory mediators, e.g., TNF-α and IL-1β, astrocyte-derived TG2 can contribute to ECM rearrangement, and possibly subsequent scar formation (Espitia Pinzón et al., 2017a). Inflammatory activation of astrocytes might therefore be necessary to reveal an effect of astrocyte-derived TG2 on OPC maturation. Alternatively, microglial-derived TG2 may play a role in OPC proliferation/myelination, as recently shown (Giera et al., 2018). It should be noted, however, that in the study by Giera et al. (2018) the presence of TG2 in microglia *in vivo* was not visualized which is important as we only observed TG2 in astrocytes in the mouse cuprizone model (Espitia Pinzón et al., 2017b) and less TG2 protein in cultured rat microglia than in astrocytes (Espitia Pinzón et al., 2017a). Of note, upon hTG2 expression, TG2 is not overexpressed, indicating that astrocytes reduced their endogenous TG2 levels. Therefore, to firmly conclude that astrocyte-derived TG2 does not affect OPC differentiation or myelination, downregulation of astrocytic TG2 should be considered.

As proof of principle, we observed that, when adding a relatively high dose of exogenous TG2 to our cultures, this affected OPC maturation. More specifically, a single exposure of OPCs to TG2 accelerated their subsequent differentiation and the extent of axonal myelination is increased in TG2-treated DRGN-OPC co-cultures. In contrast, continuous exposure of OPCs to TG2 decreased OPC differentiation and myelin membrane formation. Of relevance, microglia-derived TG2 promotes OPC proliferation and remyelination in the presence of laminin via adhesion G-protein coupled receptor (ADGRG1) signaling (Giera et al., 2018). While laminin is absent in monocultures, DRGNs express laminin, and therefore, it is tempting to suggest that the opposite effect of continuous TG2 treatment in mono- and co-cultures may be due to DRGN-derived laminin. In fact, the number of cells appeared increased upon gpTG2 addition, indicating that DRGN-derived laminin and exposure to TG2 may increase OPC proliferation, resulting in more mature myelinating OLGs at the endpoint of myelination. Also, endogenous TG2 overexpression in OPCs enhanced the percentage of MBP-positive cells at early OPC differentiation; while the percentage of MBP-positive cells of TG2-overexpressing and MOCK-transduced cells were similar at the endpoint of OPC differentiation. Accordingly, TG2-overexpressing OPCs did not affect the number of myelinated axons in DRGN-OPC co-cultures at the endpoint of myelination. This indicates that endogenousTG2 is involved in the timing of OPC differentiation. These findings are consistent with our previous observation that when TG2 activity is pharmacologically inhibited, the differentiation of OPCs into myelin-forming OLGs is dramatically delayed (van Strien et al., 2011a). Co-labeling studies revealed that TG2 immunoreactivity was lacking in Olig2-positive OPCs/OLGs in chronic active and in remyelinating MS lesions. Howeover, in the developing human cerebellum, TG2 was present in PDGFRα-positive OPCs, while we never observed cellular TG2 immunoreactivity in adult NAWM, apart from blood vessels (van Strien et al., 2011c, 2015; Chrobok et al., 2019). These data suggest that in human brain TG2 expression is transiently present in OPCs, during physiological, developmental differentiation and not in OPCs during adulthood, irrespective of the presence of inflammatory activity. Of relevance, *in vitro* studies indicate that TG2 protein and activity is absent in mature OLGs (van Strien et al., 2011a) which may be part of normal brain development. Hence, it is tempting to suggest that finding means to increase TG2 expression in OPCs may trigger their differentiation in MS lesions. On the other hand, it is good to take into consideration that TG2 is not essential for OPC differentiation, as remyelination does proceed, although delayed, as shown in TG2 –/– mice (van Strien et al., 2011a).

Increased TG2 expression is often detected in cells under pathological conditions in which inflammation is involved (Kim, 2006; van Strien et al., 2011b; Espitia Pinzón et al., 2017a). In line with this the pro-inflammatory cytokines IFN-γ and to a lesser extent TNF-α significantly increased TG2 expression in OPCs. In IFN-γ -treated OPCs, the increased TG2 expression sustained during OPC differentiation in the absence of IFN-γ. Although sustained TG2 overexpression in OPCs markedly accelerated OPC differentiation, OPC differentiation was only slightly enhanced in IFN-γ and TNF-α-treated OPCs. In contrast, IL-1β treatment of OPCs had no effect on TG2 expression, while OPC differentiation was not significantly increased. IL-1β -induced OPC differentiation has been described by others (Vela et al., 2002) though inhibition of OPC maturation has also been reported (Xie et al., 2016). Our results therefore revealed no apparent relationship between modulation of TG2 expression by pro-inflammatory cytokines and early OPC differentiation, i.e., in immature OLGs. This may be a result of activation of a more prominent TG2-independent pathway upon stimulation with IFN-γ and TNF-α or their interference with signaling pathways downstream of TG2.

IFN-γ, TNF-α, and IL-1β are pro-inflammatory cytokines that are present in MS lesions (Vartanian et al., 1995; Arnett et al., 2001; Mason et al., 2001; Codarri et al., 2010). In astrocytes and microglia, TG2 can be induced by different inflammatory mediators, including combinations of cytokines, such as IFN-γ in combination with TNF-α or IL-1β and TNF-α in combination with IL-1β (Monsonego et al., 1997; Takano et al., 2010; van Strien et al., 2011b; Espitia Pinzón et al., 2017a). In monocytes, IFN-γ is also an inducer of TG2 production (Mehta et al., 1985, 1987). Recent observations showed that of a range of cytokines applied to human monocytes, IL-4 was actually the most prominent one to induce TG2 expression in human monocytes and macrophages (Gratchev et al., 2005; Yamaguchi et al., 2016; Sestito et al., 2017). In OPCs, however, we found no increase in TG2 expression after treatment with IL-4 (data not shown, *n* = 3). Also, various combinations of IFN-γ with TNF-α or IL-1β had no additional effect on TG2 production in OPCs (data not shown). The responsiveness of glial cells to various inflammatory mediators can be explained by the presence of cytokine receptors (Cannella and Raine, 2004) and inflammatory factor-related response elements in the promotor region of the TG2 gene (Ikura et al., 1994; Kuncio et al., 1998; Gundemir et al., 2012), including NF-κB (nuclear factor-kappa B), a transcription factor involved in the regulation of expression of many inflammatory mediators (Ientile et al., 2015). The transcription of TG2 is therefore differently regulated during inflammation in these glial cells and represents different functions of these cells during inflammation. Monocyte-, macrophage- and astrocyte-derived TG2 is involved in various processes contributing to inflammation, such as adhesion and extravasation, monocyte differentiation into macrophages, efferocytosis and astrogliosis (Hostenbach et al., 2014; Chrobok et al., 2016; Espitia Pinzón et al., 2017a). TG2 in OPCs could therefore have a different function, which is not involved in processes contributing to inflammation but rather in intracellular processes that contribute to their differentiation. Our previous observations indicate that TG2 affects RhoA GTPase activity (van Strien et al., 2011a). Rho GTPases are essential in cytoskeleton rearrangements, and consequently the outgrowth of OLG processes (Czopka et al., 2009). Alternatively, TG2 can signal through ADGRG1, in OPCs in the presence of the ECM protein laminin. Signaling by TG2/laminin to ADGRG1 on OPCs improved remyelination in two murine models of demyelination (Giera et al., 2018). Interference of inflammatory mediators with one of these pathways may lead to the observed uncoupling of TG2 expression and OPC differentiation (Geras-Raaka et al., 1998; Surin et al., 2002).

Taken together, our data indicate that TG2 transiently appears in developing OPCs and promotes early OPC differentiation, while its absence in OPCs in MS lesions, irrespective of the presence of inflammatory activity, may contribute to remyelination failure (Franklin and ffrench-Constant, 2008; Kuhlmann et al., 2008). We therefore propose that development of means that stimulate TG2 expression and function in OPCs may represent a novel therapeutic strategy to improve differentiation of OPCs in MS and thereby contribute to remyelination.

## Data Availability

The datasets generated for this study are available on request to the corresponding author.

## Author Contributions

A-MvD and WB designed and supervised the project with input from BD. WB, JCdJ, NEP, HvM, JJB, and JGB conducted the described experiments. NEP and WB carried out the statistical analysis. NEP, A-MvD, and WB interpreted the data and prepared the manuscript. All authors read and approved the final version of the manuscript.

## Conflict of Interest Statement

The authors declare that the research was conducted in the absence of any commercial or financial relationships that could be construed as a potential conflict of interest.
