# Supplementary material for: Tissue Transglutaminase Promotes Early Differentiation of Oligodendrocyte Progenitor Cells
Source: Front Cell Neurosci. 2019 Jul 2;13:281. doi: 10.3389/fncel.2019.00281 (PMC6614186; doi:10.3389/fncel.2019.00281)
Supplement: Supplementary file 1 [file Data_Sheet_1.PDF]

Tissue Transglutaminase promotes early differentiation of oligodendrocyte progenitor cells

Supplementary Material

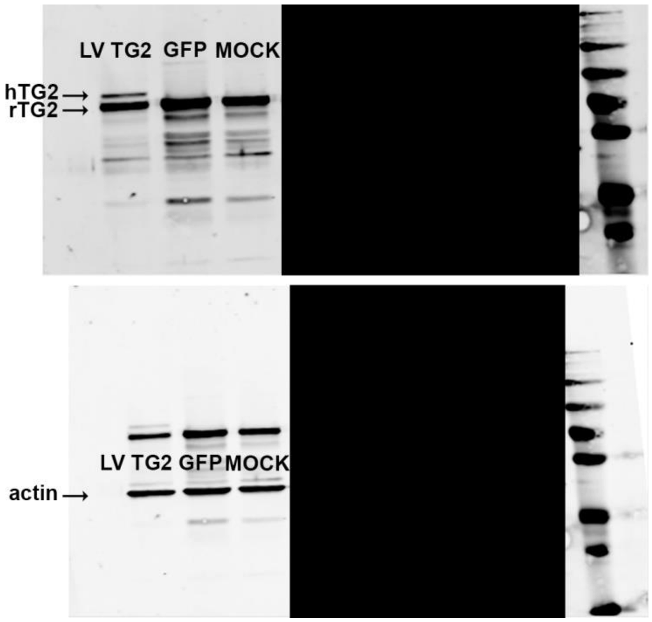

Supplementary Figure 1. Full scans of the entire original gel for Figure 1A. The top scan shows staining for TG2 after wild-type human TG2 (LV TG2), GFP and empty vector (MOCK) were lentivirally expressed in astrocytes and subjected to western blot analyses. Human TG2 (hTG2) is visible together with the endogenous rat TG2 (rTG2). The bottom scan shows the same original gel stained for actin after it was stained for TG2. Non-relevant conditions are covered with black bars.

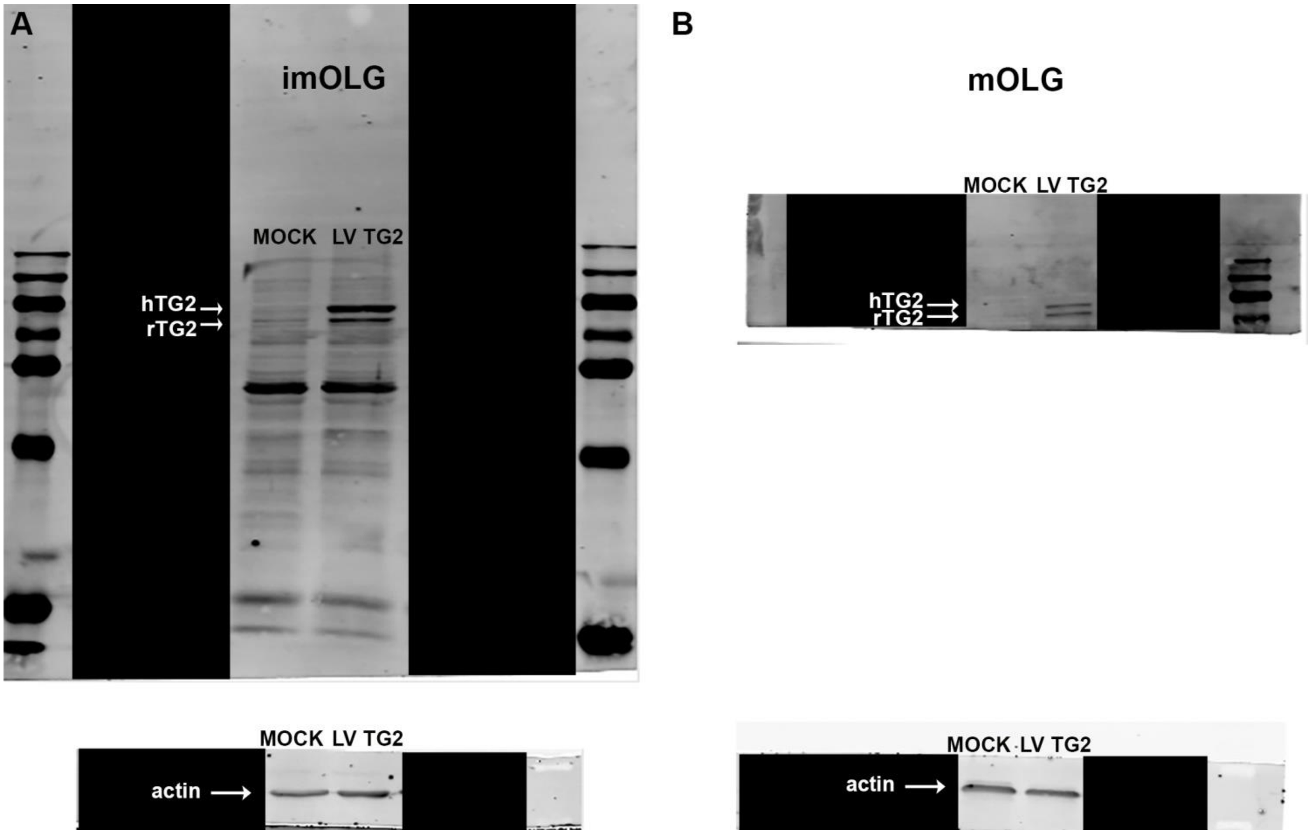

Supplementary Figure 2. Full scans of the entire original gels for Figure 5A and 5B. The top scans shows staining for TG2 after empty vector (MOCK) and wild-type human TG2 (LV TG2) were lentivirally expressed in immature (**A**) and mature oligodendrocytes (**B**) (imOLG and mOLG, respectively) and subjected to western blot analyses. Human TG2 (hTG2) is visible together with the endogenous rat TG2 (rTG2). The bottom scans show the same original gels stained for actin. Non-relevant conditions are covered with black bars.

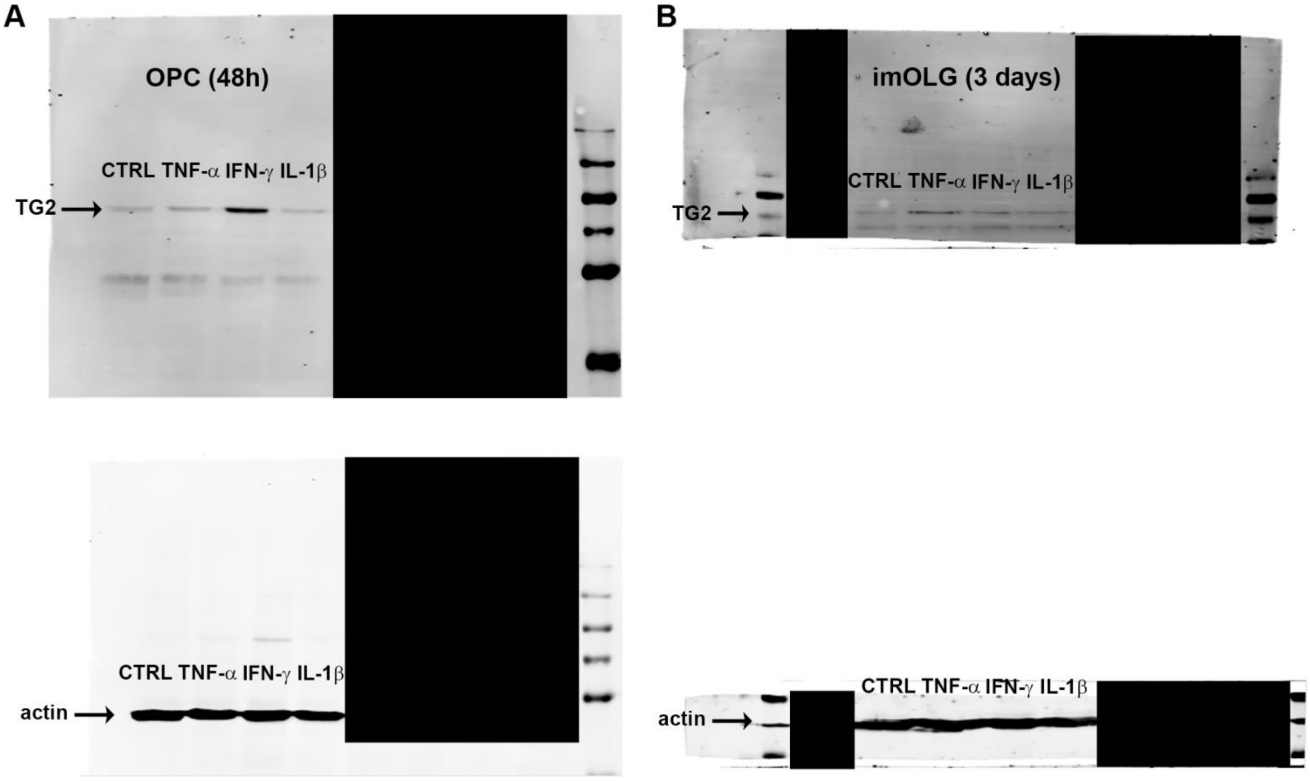

Supplementary Figure 3. Full scans of the entire original gels for Figure 7B (**A**) and 7D (**B**). The top scans shows staining for TG2 after oligodendrocyte progenitor cells (OPCs) were left untreated (CTRL) or treated for 48 hours with IFN-γ, TNF-α or IL-1β and subjected to western blot analyses (**A**) or allowed to differentiate for 3 days to immature oligodendrocytes (imOLG) in the absence (**B**) of these cytokines and subjected to western blot analyses. The bottom scans show the same original gels stained for actin. Non-relevant conditions are covered with black bars.
